# Deregulation of Autophagy and Apoptosis in Patients with Myelodysplastic Syndromes: Implications for Disease Development and Progression

**DOI:** 10.3390/cimb45050263

**Published:** 2023-05-08

**Authors:** Georgia Tsekoura, Andreas Agathangelidis, Christina-Nefeli Kontandreopoulou, Angeliki Taliouraki, Georgia Mporonikola, Maria Stavropoulou, Panagiotis T. Diamantopoulos, Nora-Athina Viniou, Vassiliki Aleporou, Issidora Papassideri, Panagoula Kollia

**Affiliations:** 1 Division of Genetics & Biotechnology, Department of Biology, National and Kapodistrian University of Athens, 15772 Athens, Greece; gtsekour@biol.uoa.gr (G.T.); agathan@biol.uoa.gr (A.A.);; 2 Hematology Unit, First Department of Internal Medicine, Laikon General Hospital, National and Kapodistrian University of Athens, 11527 Athens, Greece; 3 Division of Cell Biology and Biophysics, Department of Biology, National and Kapodistrian University of Athens, 15772 Athens, Greece

**Keywords:** myelodysplastic syndromes, autophagy, apoptosis, gene expression profiling

## Abstract

(1) Background: Myelodysplastic neoplasms (MDSs) consist of a group of blood malignancies with a complex biological background. In this context, we investigated the role of autophagy and apoptosis in the pathogenesis and progression of MDSs. (2) Methods: To address this issue, we performed a systematic expression analysis on a total of 84 genes in patients with different types of MDSs (low/high risk of malignancy) versus healthy individuals. Furthermore, real-time quantitative PCR (qRT-PCR) was used to validate significantly upregulated or downregulated genes in a separate cohort of MDS patients and healthy controls. (3) Results: MDS patients were characterized by lower expression levels for a large series of genes involved in both processes compared to healthy individuals. Of importance, deregulation was more pronounced in patients with higher-risk MDS. Results from the qRT-PCR experiments displayed a high level of concordance with the PCR array, strengthening the relevance of our findings. (4) Conclusions: Our results indicate a clear effect of autophagy and apoptosis on MDS development, which becomes more pronounced as the disease progresses. The results from the present study are expected to assist in our understanding of the biological background of MDSs as well as in the identification of novel therapeutic targets.

## 1. Introduction

Myelodysplastic neoplasms (MDSs) comprise a heterogeneous group of clonal hematopoietic stem cell neoplasms of uncertain etiology. MDSs are primarily a disease group of the elderly, with the mean age of patients at diagnosis being 65 years [1]. According to the most recent data from the SEER (Surveillance, Epidemiology, and End Results) Program Registry, the incidence of MDSs is 4.9 per 100,000 people per year, with the rate increasing with age [2,3].

The onset and clinical course of MDSs are very heterogeneous; specifically, some patients are asymptomatic or present with mild clinical symptoms at diagnosis while exhibiting an overall slow rate of progression (if any). In contrast, other patients experience an aggressive disease that may lead to death within 6 months from the initial diagnosis [4]. From an ontogenetic standpoint, MDSs are divided into: (i) primary (de novo), which do not have a clearly defined etiology, and (ii) secondary or related to myelotoxic therapy (therapy-related MDS, t-MDS) that occur after the application of chemotherapy or radiation [5].

Eight different patient classification systems have been designed over the years, based on morphological and/or prognostic parameters [5,6]. Nowadays, the Revised International Prognostic Scoring System (IPSS-R), which is based on the peripheral blood (PB) cell counts, marrow blast percentage, and cytogenetic aberrations, represents the most widely used classification system for patients with MDSs [7]. Furthermore, it is worth mentioning that a new classification system was published in 2022, the IPSS-Molecular (IPSS-M), incorporating multiple high-risk gene mutations into the previously established cytogenetic and clinical parameters [8]. Of importance, the IPSS-M score presents with higher predictive accuracy, especially for aged patients [9].

At the cellular level, MDSs are characterized by abnormal maturation and differentiation of the hematopoietic cells, as well as inefficient hematopoiesis. Overall, this deregulation results in morphological and functional cell defects together with an increased risk of transformation to secondary acute myeloid leukemia (AML) [10]. Yet, the exact pathogenetic mechanisms underlying the onset and evolution of MDSs remain largely unknown; several independent studies have focused on the investigation of the pathophysiology of these neoplasms at different levels [11]. At the genetic level, a wide range of recurrent gene mutations have been documented in MDS patients, being implicated in specific processes/mechanisms, such as epigenetic regulation, gene transcription, and DNA repair [12].

Furthermore, cellular processes, such as cell differentiation and signaling, have also been causally implicated in the development and/or progression of MDSs [13]. More specifically, (macro)autophagy and apoptosis have already been established as critical for the development of MDSs [14,15,16]. These two cell mechanisms are closely linked; components of the intrinsic and extrinsic apoptotic pathways engage with autophagy regulators to dictate cell fate in multiple different ways [17]. Furthermore, autophagy and apoptosis can also regulate each other, either through the autophagic degradation of apoptotic proteins or, alternatively, via the cleavage of autophagic proteins by caspases [17]. In detail, some of the most characteristic examples include BECN1-BCL2 interactions [18], caspase-mediated BECN1 cleavage [19], and UVRAG-BAX interactions [20]. Thus, deregulation in autophagy and apoptosis could have a combinatorial and, thus, enhanced impact on the pathology of MDSs.

The regulation of apoptosis is known to be abnormal in MDSs; specifically, ineffective hematopoiesis resulting in PB cytopenias is a hallmark of the disease, while excessive apoptosis of hematopoietic precursors in the bone marrow (BM) appears to be one of the underlying mechanisms [21]. Numerous studies over the past decade have shown that BM mononuclear cells from patients with early-stage MDSs are prone to apoptosis [22]. On the other hand, BM cells in advanced stages of MDSs show less apoptotic potential while also exhibiting features related to a high proliferative signature [22]. However, these findings are still not well understood, i.e., how disease evolution may transform the BM cells from MDS patients from apoptosis sensitive to resistant.

In addition, impairments in various signaling pathways may lead to the deregulation of autophagy in MDSs [16]. Findings from multiple studies further support the notion that the pathogenesis of MDSs is driven by abnormalities in the process of autophagy [23]. In detail, changes in the autophagic motor of hematopoietic progenitor cells, combined with the accumulation of additional molecular changes, may eventually lead to the malignant transformation observed in MDSs [16]. This is particularly evident in patients with low-risk MDSs, i.e., patients who are less likely to progress to AML, where high levels of erythroid cell autophagy have been documented during the final differentiation stage [24]. Defects in autophagy were also evident in a study in mice, reporting deficiencies in the *ATG7* gene in hematopoietic stem cells (HSCs); these defects may eventually lead to an increased proliferation rate for cells belonging to the marrow lineage similar to that observed in high-risk MDSs [25].

In addition to the obvious benefits, all aforementioned studies were mainly focused on the study of a limited number of genes involved in either apoptosis or autophagy, thereby hindering the robust assessment of the actual impact of these processes on MDS pathogenesis. To this end, we systematically assessed the expression levels of a large series of genes involved in autophagy and apoptosis in samples from MDS patients and healthy individuals. Experiments showed downregulation in a series of genes involved in both processes in MDSs compared to healthy donors, which was stronger in higher-risk patients. These findings allude to the existence of defects in autophagy and apoptosis that become more pronounced along the natural history of MDSs.

## 2. Materials and Methods

### 2.1. Study Group

A total of 34 patients with MDSs with a median age of 75 years (range: 59–90 years) were included in the study, together with 23 healthy donors with a median age of 55 years (range: 24–72 years). All patients were diagnosed with MDSs based on the 2016 World Health Organization (WHO) classification criteria and had not received treatment before the sampling procedure. All experiments performed in the study involving human samples were reviewed and approved by the Scientific Committees of all collaborating institutes (No. 2/2022) in accordance with the 1964 Helsinki Declaration and its later amendments or comparable ethical standards. Experiments were undertaken with the understanding and written informed consent of all participants.

Classification of MDS patients was performed according to IPSS-R scoring system [7]. At the time of investigation, 2 MDS patients were defined as very low-risk score (≤1.5 points), 11 with low-risk score (>1.5–3 points), 15 patients with high-risk (>4.5–6 points), and 6 patients with very high-risk score (>6 points). For the purposes of the present study, MDS patients were divided into 2 main categories: those with an IPSS-R score of ≤3.5 (very low, low, or intermediate risk) were classified into the lower-risk category, while patients with an IPSS-R score of >3.5 (high and very-high risk) were grouped into the higher-risk category. Bone marrow samples were obtained from all patients analyzed in the study, while PB samples were collected from all healthy controls as well as for 3 MDS patients. Basic clinical and biological information regarding our MDS patient cohort is summarized in Table 1 and Supplementary Table S1.

### 2.2. Cell Separation

Mononuclear cells were isolated from all participants using Ficoll-paque (GE Healthcare, Bio-Sciences AB, Uppsala, Sweden) and pelleted after hypotonic lysis of erythrocytes and two washes in phosphate-buffered saline (PBS tablets) (Calbiochem, Merck KGaA, Darmstadt, Germany y).

### 2.3. RNA Extraction

Total RNA was extracted using the TRIZOL reagent (Invitrogen, Carlsbad, CA, USA) following the manufacturer’s instructions.

### 2.4. Real-Time PCR-Based Array Analysis

Total RNA (0.5 μg) was extracted and used as template for cDNA synthesis in 10 patients with MDSs (5 lower-risk, 5 higher-risk) and 3 healthy controls using the RT^2^ First Strand Kit. The Human Autophagy RT^2^ Profiler™ PCR Array (Qiagen GmBH, Hilden, Germany) was used to screen a panel of 84 genes related directly or indirectly to the processes of autophagy and apoptosis. In more detail, the panel contained: (i) 45 genes directly linked to autophagy and (ii) 39 genes indirectly related to the autophagic process. Among them, 33 genes are implicated in the process of apoptosis and 6 genes are related to cell cycle regulation. Gene categorization according to function is given in Supplementary Table S2. More information regarding the experimental process can be found in the Supplementary Material.

### 2.5. Non-Supervised Clustering Analysis

Non-supervised hierarchical clustering analysis was performed using RT^2^ Profiler PCR Array Data Analysis Webportal. The magnitude of gene expression was used as a distance/dissimilarity metric. In more detail, the magnitude of gene expression was determined by calculating the 2^−ΔCT^ for each individual gene. In terms of linkage method, the “Average” linkage was chosen; this option defines the distance between clusters using the average of the differences in the distance/dissimilarity metric between all pairs of genes among the different clusters. Cell colors represent the relative magnitude of gene expression: the black color represents the average magnitude of gene expression, the brightest green represents the smallest value, while the brightest red represents the highest value.

### 2.6. Quantitative Real-Time PCR (qRT-PCR)

Real-time quantitative PCR (qRT-PCR) was used to validate significantly upregulated or downregulated genes (fold changes of >2 or <−2, respectively) in a separate cohort of 24 MDS patients and 20 healthy controls. cDNA was synthesized using the Superscript II reverse transcriptase (Invitrogen, Carlsbad, CA, USA). PCR amplification was performed using the Hot Firepol EvaGreen Taq polymerase (Solis Biodyne, Tartu, Estonia) on a PikoReal^TM^ Real-Time PCR System (Thermo Fisher Scientific, Applied Biosystems, Waltham, MA, USA). Gene expression levels were normalized against those of the housekeeping gene *HPRT1* using the 2^−∆∆CT^ method. All samples were run in duplicate. Supplementary Table S3 summarizes all primer sequences used in the context of the qRT-PCR experiments, while Supplementary Table S4 shows the standard deviation values between sample replicates.

### 2.7. Western Blotting

Proteins were extracted from patient and control cells using a radioimmunoprecipitation assay buffer (Thermo Fisher Scientific, MA, USA) containing protease and phosphatase inhibitors (Thermo Fisher Scientific, MA, USA). Protein concentration of the extracts was determined by using a Bicinchoninic Acid (BCA) Protein Assay Kit (Thermo Fisher Scientific, MA, USA). Equal amounts (25 µg) of protein were separated via 10~12% SDS-PAGE and blotted onto PVDF membranes, which were blocked with 5% non-fat milk at room temperature for 1 h. Subsequently, the membranes were incubated with specific primary antibodies against TGM2 (1:1000; cat. no. MA5-12739, Thermo Fisher Scientific, MA, USA), BCL2 (1:1000; cat. no. sc-7382, Santa Cruz Biotechnology, Santa Cruz, VA, USA ), LC3B (1: 500; cat. no. 3868, Cell Signaling Technology, Beverly, MA, USA), and GAPDH (1:1000; cat. no. E-AB-20079, Elabsience, Houston, Texas, USA) overnight at 4 °C. The next day, the blots were probed with secondary goat anti-mouse (1:1000; cat. no. E-AB-1008, Elabscience, TX, USA) or goat anti-rabbit antibodies (1:1000; cat. no. E-AB-1008, Elabscience, TX, USA) at room temperature for 1 h. The blots were visualized using an ECL system (Thermo Fisher Scientific, MA, USA) and analyzed via Image J. Experiments were repeated three times for each sample.

### 2.8. Data Accessibility

The data generated in the context of the study are openly available in GEO database at [https://www.ncbi.nlm.nih.gov/geo/query/acc.cgi?acc=GSE204797], reference number (GSE204797), accessed on 26 May 2022.

### 2.9. Statistical Analysis

The resulting threshold cycle values (CT) were analyzed using the data analysis webportal at http://www.qiagen.com/geneglobe, accessed on 26 May 2022. The data analysis web portal calculates fold change/regulation using the delta CT method. Fold change is then calculated using 2^−ΔCT^ formula. The student’s *t*-test was used to compare gene expression levels between groups of MDS patients and healthy controls (*p* < 0.05) according to the manufacturer’s instructions. Specifically, *p* values were calculated based on a student’s *t*-test of the replicate 2^−ΔCT^ values for each gene in the MDS patient and control groups. The data analysis web portal was also used for the generation of scatter plots, volcano plots, and heat maps. To examine potential differences in the distribution of genes showing distinct expression levels among the formed clusters (after the application of non-supervised clustering), the chi-square test was applied. For these comparisons, a significance level of *p* = 0.05 was set.

## 3. Results

### 3.1. Identification of Different Expression Pattern of Autophagy and Apoptosis Associated Genes in Patients with MDSs

#### 3.1.1. Downregulation of Genes Related to Autophagy and Apoptosis in Patients with MDSs

**Figure 1:**
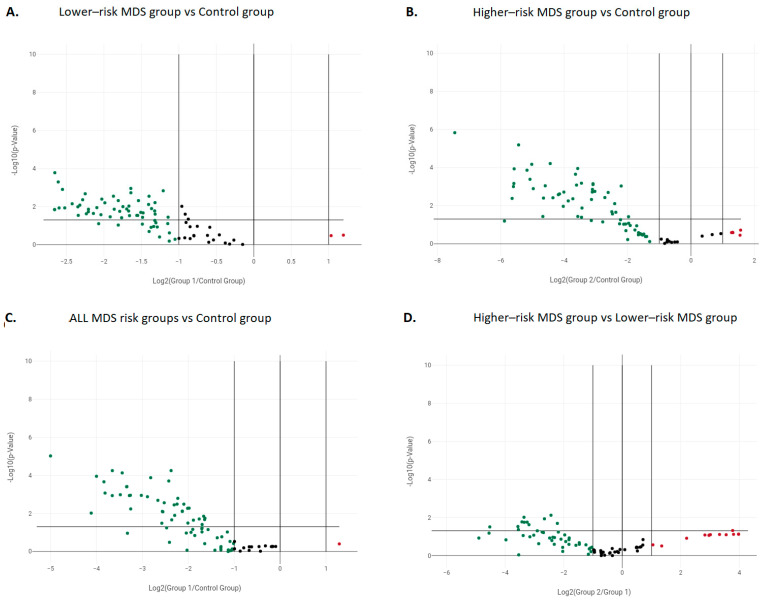
Expression levels of genes implicated in the processes of autophagy and apoptosis in MDSs versus healthy donors. The volcano plots depict significant gene expression changes by plotting the log2 of the fold changes in gene expression on the *x*-axis versus their statistical significance on the *y*-axis. The center vertical line indicates unchanged gene expression, while the two outer vertical lines indicate the selected fold regulation threshold. The horizontal line indicates the selected *p*-value threshold. Genes with data points in the far upper left (green dots) are downregulated, while those at the far upper right (red dots) are upregulated according to the selected fold regulation and *p*-value thresholds. Differences in the expression of autophagic and apoptotic genes between: (**A**) all MDS samples compared to the control group, (**B**) the lower-risk MDS group and the control group, (**C**) the higher-risk MDS group and the control group, (**D**) the higher-risk and lower-risk MDS groups.

As a first step, we compared the expression profiles of genes associated with the autophagy and apoptosis pathways in 10 patients with MDSs, irrespective of disease risk, against 3 healthy donors. In total, 39/84 (46.4%) genes displayed a significant downregulation in MDSs versus healthy controls (*p* < 0.05) (Supplementary Table S5, Figure 1A). Of those, 20/45 genes (44.4%) were implicated in the process of autophagy, involving both the formation and elongation of the autophagic vesicle as well as its subsequent fusion with the lysosome. At the individual gene level, the genes showing the strongest downregulation in MDSs compared to healthy controls were the following: (i) *CTSS*, which belongs to the family of lysosomal cysteine proteases and is involved in lysosomal protein degradation, (ii) *GABARAP*, *GABARAPL2,* and *RGS19*, implicated in autophagic vesicle formation, (iii) *HSPA8*, related to chaperone-mediated autophagy, and (iv) *SQSTM1*, involved in the lysosomal degradation of misfolded/unfolded proteins. In contrast, the *TGM2* gene was the sole autophagic gene showing overexpression in MDSs versus the control group.

Furthermore, 16/34 genes (47.1%) related to apoptosis and 4/6 genes (66.7%) linked to the regulation of the cell cycle were found to be significantly downregulated in MDS samples versus healthy controls. Among apoptotic genes, the *TNFSF10*, *CASP8*, *FAS,* and *BID* exhibited the lowest levels of expression in MDSs versus healthy controls. Concerning genes related to the regulation of the cell cycle, the *TGFB1* and *PTEN* genes displayed the highest level of downregulation.

Due to the significant age difference between the median ages of the MDS patients and healthy controls (75 versus 55 years, respectively), we divided healthy controls into two groups: (i) a younger group under 55 years of age (*n* = 6) and (ii) a group of older individuals of 55 years and over (*n* = 14); we then performed a comparative analysis of their mRNA expression profiles. Of importance, the gene expression profiles of the younger and older control groups were highly similar.

Subsequently, we compared the gene expression profile of each MDS group (lower-risk and higher-risk MDS) against that of the control group (Supplementary Table S5). Overall, a higher number of genes were found to be downregulated in lower-risk MDS samples (51/84, 60.7%) than in samples from the higher-risk group of MDS patients (42/84, 50%) when compared against the control group (Figure 1B and 1C, respectively) as well as against each other (Figure 1D). Twenty-five genes (29.8%) were found to be significantly downregulated (*p* < 0.05) in both groups of MDS, perhaps alluding to a stronger relevance of apoptosis in MDS development. In terms of function, this gene set was characterized by an enrichment of genes implicated in apoptosis; more specifically, shared downregulated genes included: (i) 11/45 genes (24.4%) related to autophagy, (ii) 12/33 genes (36.4%) implicated in apoptosis, and (iii) 2/6 genes (33.3%) involved in cell cycle regulation. Finally, the autophagic *TGM2* gene was upregulated in both groups of MDSs compared to healthy controls; in detail, fold change compared to controls was 2.04 in lower-risk MDS and 2.32 in higher-risk MDS, respectively.

With regard to genes being significantly downregulated only in the comparison between lower-risk MDSs and controls, 14/44 genes (31.8%) belonged to autophagy, 12/34 genes (35.3%) were implicated in apoptosis, and, finally, 1/6 genes (16.7%) were assigned to the regulation of the cell cycle. The respective numbers of genes significantly downregulated in higher-risk MDS versus controls were 10 in autophagy (22.7%), 6 in apoptosis (17.6%), and 1 in cell cycle control (16.7%), respectively.

Next, we assessed the level of gene expression downregulation in both MDS groups following a more quantitative approach, i.e., by calculating the median and average values of fold change for all genes being significantly deregulated in each group of patients. According to the results, downregulated genes in higher-risk MDS showed more pronounced fold changes compared to the healthy donors (median: −16.3, mean: −28.3) than downregulated genes in lower-risk MDS (median: −3.5, mean: −3.8).

#### 3.1.2. Higher-Risk MDS Has a Distinct Autophagic Gene Expression Signature Compared to Lower-Risk MDS and Control Samples

**Figure 2:**
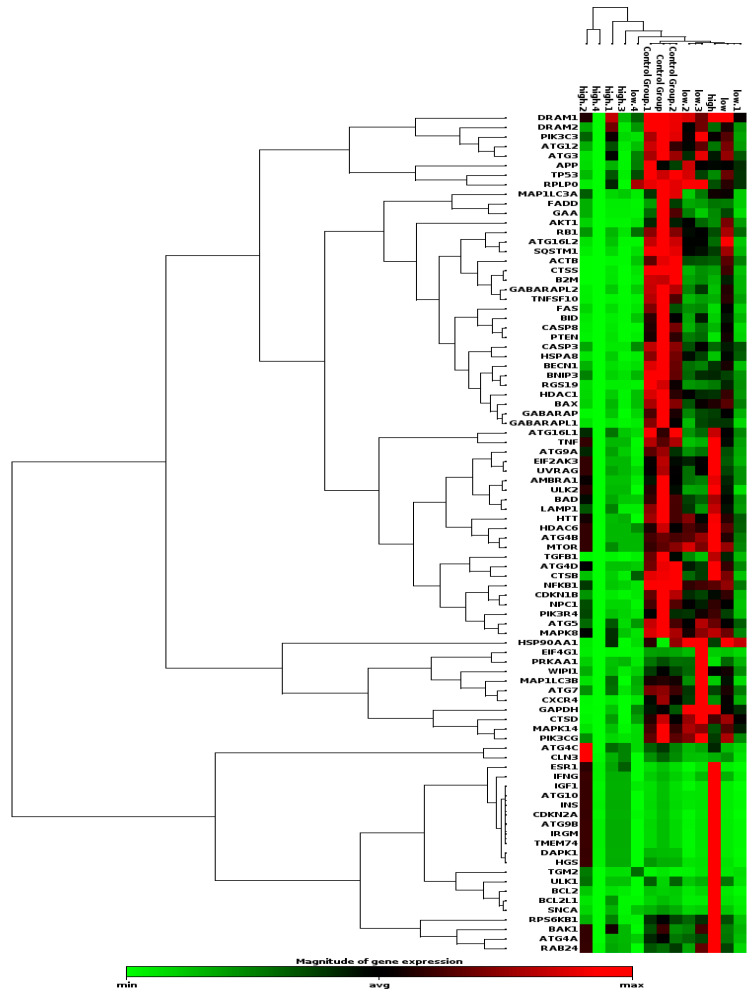
Heatmap of unsupervised hierarchical clustering based on the PCR array gene expression data of low-risk and high-risk MDS, as well as control samples. Columns represent samples and rows represent genes, respectively. The color bar indicates mRNA expression levels (bright red indicates high expression; black indicates average expression; bright green indicates low expression).

Non-supervised hierarchical clustering analysis, depicted as a heat map in Figure 2, led to the formation of a large cluster containing the majority of analyzed samples (8/13 samples, 61.5%). Of importance, this cluster was enriched in samples belonging to the lower-risk MDS (4/5 samples, 80%) and healthy control groups (3/3 samples, 100%). In contrast, only a single higher-risk sample was assigned to this cluster. The remaining samples, corresponding to one lower-risk MDS and four higher-risk MDS cases formed smaller, more distant clusters.

When looking at the patterns of gene expression within each cluster, the aforementioned large cluster (corresponding mostly to lower-risk MDS and control samples) was characterized by overall higher levels of gene expression compared to all other clusters corresponding to higher-risk MDS cases, indicating a higher level of deregulation in the pathways of autophagy and apoptosis in the latter MDS group. Intriguingly, there was a small gene set (*n* = 23 genes) with consistently low expression levels in all clusters, comprising genes implicated in both the processes of autophagy and apoptosis. A slight enrichment was observed for the latter (9/33 genes, 27.3%) versus the former (11/45, 24.4%), yet it was not statistically significant.

Overall, this analysis indicates that high-risk MDS displays a distinct expression profile of genes implicated in autophagy and apoptosis compared to low-risk MDS and healthy donors, while also showing that the most prominent gene expression differences between the two MDS risk groups concern the process of autophagy.

### 3.2. Downregulation of Genes Related to Autophagy and Apoptosis in MDS Is Corroborated by qRT-PCR

In order to substantiate the results from the analysis of the PCR array data, we analyzed the expression levels of a number of key genes related to the autophagy and apoptosis processes using qRT-PCR. The criteria for gene selection concerned gene function and expression patterns obtained by the RT^2^ Profiler PCR Array. In more detail, 10 genes implicated in different stages of autophagy were selected, such as autophagosome initiation (namely, *AMBRA1, PI3KC3,* and *UVRAG*), phagophore elongation (namely, *ATG5*, *ATG12,* and *ATG16L1*), and phagophore maturation (*DRAM1*, *DRAM2,* and *MAP1LC3B*), all of which exhibited significant expression differences between at least the MDS risk group and the control group. Furthermore, we also selected the *TGM2* gene, given that this was the only gene exhibiting overexpression in MDS samples versus the control group. Moreover, the expression of five genes related to apoptosis was also assessed, including four genes showing a significant overexpression in MDS versus the control group according to the PCR array results (namely, *BCL2*, *CASP3*, *CASP8,* and *CTSB*), as well as *CASP7*. This type of analysis was performed in a larger, independent series of samples, consisting of 24 MDS patients (14 with low-risk MDS and 10 with high-risk MDS), as well as 20 healthy controls.

**Figure 3:**
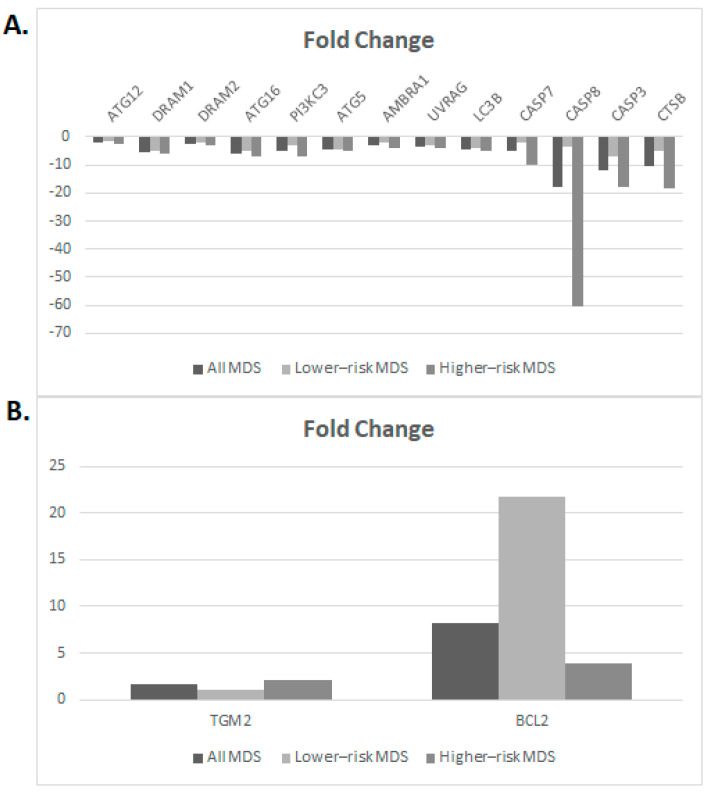
Gene expression changes for a selected group of 15 genes in MDSs compared to healthy controls using qRT-PCR. Bar plots depict the gene expression levels of: (**A**) 13 downregulated genes and (**B**) 2 upregulated genes in different MDS groups (all MDS, low-risk and high-risk MDS, respectively) compared to healthy controls. The *HPRT1* gene was used as internal control.

Given the different sample sources between MDS patients and healthy controls (BM vs. PB), we also assessed the impact of this factor through a comparative mRNA expression analysis of the *ATG, DRAM, AMBRA1, PI3KC3, UVRAG, TGM2, BCL2, CASP7,* and *LC3B* genes in BM and PB samples from three MDS patients. No differences were observed in the gene expression levels between samples of different origin from the same patient (standard deviation values of normalized Ct values from these experiments are given in Supplementary Table S6). This observation is in line with three previous reports [26,27,28], which indicate that PB could be used for molecular analysis. These data provide us with the ability to compare patients’ BM samples with controls’ PB samples. According to the qRT-PCR experiments, a high level of concordance with the PCR array was observed; more specifically, all but the *TGM2* and *BCL2* genes were downregulated in both types of MDSs compared to the control group (Table 2, Figure 3).

Table 2 presents the mean values for the fold change (up- or downregulated) in different groups of MDS samples compared to controls. *p*-values were estimated using the student’s *t*-test of the replicate 2^(−ΔCT) values for each gene in the patient and control groups. The *HPRT1* gene was used as an internal standard. Genes were sorted based on their function.

When considering all MDS patients as a single group, 10/15 genes (66.7%) displayed a statistically significant downregulation (*p* < 0.05) compared to the group of healthy individuals, including genes implicated in both autophagy and apoptosis (Figure 3A). Specifically, significantly lower expression levels in MDSs versus healthy controls were observed for *PI3KC3*, *UVRAG*, *ATG5*, *ATG16L1*, *DRAM1,* and *MAP1LC3B* as well as *CASP3*, *CASP8*, *CTSB,* and *CASP7*, respectively. Of relevance, the expression levels of several genes related to the autophagic process, namely the *ATG5*, *ATG12*, *ATG16L1*, *PI3KC3, AMBRA1,* and *UVRAG* genes, showed highly similar expression patterns among individual patients.

As a next step, we followed the same approach and classified MDS patients into the lower-risk and higher-risk groups and performed comparisons against the control group. This analysis showed a high level of similarity between the two MDS groups, given that the expression of 5/15 genes (33.3%), namely, *PI3KC3*, *UVRAG*, *ATG5*, *DRAM1,* and *CASP3,* was significantly downregulated in both compared to the healthy controls (Figure 3A). In regard to group-specific gene downregulation, the *ATG16L1* and *MAP1LC3B* genes showed significantly different expression levels only in lower-risk MDSs, whereas the opposite trend was observed for the genes *ATG12*, *DRAM2, CTSB,* and *CASP7*.

Furthermore, the qRT-PCR results confirmed the previously observed higher expression of *TGM2* in both the lower-risk and high-risk MDS groups, yet without statistical significance. The only case of discordance between the PCR array and qRT-PCR results concerned the *BCL2* gene; specifically, the *BCL2* mRNA levels were higher in both types of MDSs compared to the healthy donors, with the difference being statistically significant only in the lower-risk MDS group (Figure 3B). A possible explanation for this discrepancy could be that the primers used in the qRT-PCR experiments, which allowed for the detection of all splice variants of the *BCL2* gene, were not identical to those utilized in the RT^2^ Profiler Human Autophagy PCR Array.

### 3.3. mRNA Expression Levels Correlated Well with the Corresponding Protein Levels in the Cases of LC3-II, TGM2, and BCL2

**Figure 4:**
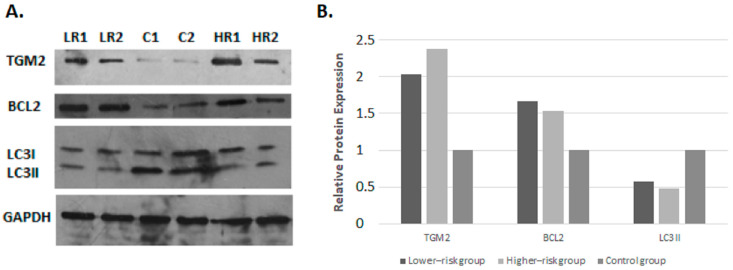
(**A**) Protein expression levels of TGM2, BCL2, and LC3B in 2 control samples (Ctrl1, Ctrl2), 2 higher-risk (H-R1, H-R2), and 2 lower-risk MDS patients (L-R1, L-R2). Analysis of protein expression levels was performed via Western blotting. The intensity of the bands was quantified with ImageJ software, and the expression ratio of each protein was estimated relative to the GAPDH reference protein expression levels. (**B**) Relative protein expression levels for TGM2, BCL2, and LC3B in each MDS risk group were determined in comparison to the control group. Experiments were repeated three times with comparable results. Our findings indicate increased expression levels for the TGM2 and BCL2 proteins as well as decreased expression levels for LC3B in MDS patients compared to healthy controls.

To validate the biological significance of the mRNA expression analysis, we performed preliminary Western blot experiments for the investigation of the protein levels of TGM2, BCL2, and LC3B in two lower-risk patients and two higher-risk MDS patients as well as in three healthy individuals. Densitometric analysis revealed elevated levels of TGM2 and BCL2 as well as a lower expression for LC3B in patient samples from both MDS groups compared to the controls (Figure 4). More specifically, the TGM2 protein levels in lower- and higher-risk MDS compared to healthy controls were 2.1- and 2.4-times higher, respectively. The respective values for BCL2 were 1.5 and 1.7 for higher- and lower-risk MDS. In contrast, the LC3B protein was 1.5- and 2-times lower in higher- and lower-risk MDS, respectively, when compared against the healthy controls. These findings were in line with the results obtained from mRNA expression analysis; more specifically, the expression differences between MDS patients and the controls for TGM2 and LC3B showed a strong correlation between the mRNA and protein levels. On the other hand, the expression difference of the *BCL2* gene between MDSs and controls was more modest at the protein level compared to the mRNA level.

## 4. Discussion

MDSs are considered to be a complex group of blood malignancies, mainly due to their elusive pathophysiology and considerable clinical heterogeneity [1]. Furthermore, current clinical classification systems have limited prognostic and predictive value, especially in low-risk patients, highlighting the necessity for additional biological markers [29]. In this context, the results from several recent studies led to the notion that deregulation of the processes of autophagy and apoptosis may be key to the pathogenesis of MDSs [30]. More specifically, the status of several genes implicated in these processes has been associated with the pathogenesis, prognosis, and therapeutic response in MDSs [31]. Furthermore, increased programmed cell death (PCD) has been recorded in a series of relevant studies, based on increased in situ DNA end labeling (TUNEL staining) [32]. However, the actual role and effect of autophagy and apoptosis in the context of MDS biology and clinical outcome have not been fully elucidated.

To this end, we carried out a systematic expression analysis in a large series of genes related to these processes in patients with MDSs with different prognoses through the application of microarray-based technology, complemented by qRT-PCR and Western blot experiments. Of relevance, our analysis was performed on BM samples from MDS patients and PB samples from healthy individuals that were also considerably younger. To address the impact of these aspects, we performed the following analyses: (i) a comparative gene expression analysis in paired BM and PB samples in three MDS patients and (ii) a comparative gene expression analysis between younger and older healthy individuals (the threshold was 55 years of age). Significant differences were not evident in any of the aforementioned cases, leading to some important conclusions. First, the overall concordance between the BM and PB mRNA expression profiles in MDSs meant that peripheral blood could be used for the study of the molecular mechanisms of these malignancies. Second, the lack of significant differences between young and older healthy individuals may mean that the impact of age, at least in terms of the autophagy and apoptosis mechanisms, may be limited.

Overall, the gene expression profile of MDS patients was clearly distinct from that of healthy individuals, being characterized by a significant downregulation of genes implicated in both the autophagic and apoptotic processes. Subsequently, our MDS cohort was classified in groups of patients with different disease prognoses: lower-risk MDS versus higher-risk MDS. Both groups were characterized by a significant downregulation of genes implicated in both processes compared to healthy individuals, further corroborating their relevance along the MDS trajectory. The downregulation in both processes was more pronounced in higher-risk MDSs, indicating their importance in the progression of the disease. This finding was further confirmed by the results of the unsupervised hierarchical clustering analysis of the data, showing a more distinct gene expression profile for higher-risk MDS compared to lower-risk MDS and healthy controls. Of interest, this difference was due to differences in the expression levels of genes related to both autophagy and apoptosis, perhaps indicating the relevance of these mechanisms in the progression of MDSs.

Focusing on autophagy, significant differences were identified between MDS patients and healthy controls in the expression of genes involved in all successive stages of autophagy [33]. Concerning the formation of the autophagosome, we observed a strong downregulation of PI3K complex genes, such as *PIK3C3*, *AKT1*, *ULK1,* and *BECN1,* in MDS patients compared to healthy controls. This finding is in line with recent evidence from molecular and cellular studies reporting abnormalities in a series of autophagic genes, such as *AMPK*, *ULK1*, *AKT*, *MTOR*, and *BECN1,* that block several steps of the autophagic process, some of which were reported as being relevant in the development of MDSs and their transformation to AML [16]. Moreover, the mRNA expression levels of the autophagy promoter genes *AMPK* and *ULK1* were also reported as significantly lower in the erythroid cells of high-risk MDS patients [23]. Additionally, a proteomic approach also revealed decreased levels of AKT and BECN1 in higher-risk MDS patients [34]. In terms of autophagy regulation, a significantly decreased expression of several *ATG* genes was identified, which probably reflects a reduced autophagy rate [35]. In more detail, a low expression of *ATG7* [36] and other autophagy regulation defects [37] in MDSs was shown to promote malignant transformation through the inhibition of autophagy. In parallel, studies in mouse models reported deficiencies in genes of the *ATG* family in HSCs that resulted in the development of a high-risk MDS phenotype [38]. Finally, a significant decrease in the expression of the autophagic indicator LC3B was observed in our MDS cohort, indicating a decreased activity of autophagy. This finding was further supported by our Western blot validation experiments, showing decreased expression of LC3B at the protein level in both MDS risk groups. Focusing on MDS, the LC3B protein expression levels were lower in higher-risk MDS, corroborating the notion that the autophagic flux may be reduced as the disease progresses. However, these findings were based on the analysis of a few samples, and larger cohorts need to be analyzed in order to fully clarify this issue. Of relevance, the expression of LC3B in GlycoA+ nucleated RBC was reported to be lower in high-risk MDS patients compared to controls [23]. A significant reduction in the expression of *DRAM1* and *LAMP1* was also observed in the MDS group, contributing to the inhibition of autophagosome–lysosome fusion.

Our results also showed that a significant fraction of apoptotic genes (including *BAD*, *BAX, BAK, BID, FAS,* and *FADD*) was significantly downregulated in MDS patients. With regard to the status of caspases, we noted that *CASP8*, *CASP3,* and *CASP7* expression levels were significantly lower in patients with MDSs versus healthy individuals. Increased CASP3 activity was noted after an ex vivo culture of BM cells from patients with MDSs, yet only a minority of MDS cases displayed actual evidence of CASP3 activation [39]. This finding, in the light of the existing evidence on MDSs, could suggest that apoptosis may be inhibited or may not be the predominant cell death pathway activated in MDSs, warranting further investigation.

Altogether, the application of qRT-PCR corroborated the data obtained through the microarray technology while also highlighting specific gene expression patterns. More specifically, a series of genes (namely, *ATG5*, *ATG12*, *ATG16L1*, *PI3KC3, AMBRA1,* and *UVRAG)* showed similar expression patterns across different MDS patients, alluding to regulation by the same factors. Of interest, the TFEB transcription factor was shown to regulate the expression of both the *ATG5* and *UVRAG* genes, given that its inactivation led to the silencing of the aforementioned autophagic genes [40]. In addition, FOXO3 was the first FOXO member identified as a transcriptional regulator of the *ATG12* and *PI3KC3* genes [41]. Having said that, the regulatory roles of these transcription factors should be further investigated by integrating data from epigenetics and protein–protein interactions in order to be established. The only difference between the results obtained through the different methodologies employed in the present study concerned the *BCL2* gene, which was found to be significantly upregulated in MDSs versus healthy controls. BCL2 overexpression at the protein level was confirmed in both MDS risk groups through immunoblotting experiments. Yet, the level of overexpression was much lower compared to the mRNA level, perhaps alluding to the existence of other mechanisms regulating the levels of BCL2 post-transcription. High expression levels of BCL2 have been shown to lead to the inhibition of both autophagy- and apoptosis-associated cell death [42]; specifically, BCL2 binds to the BAK/BAX complex, therefore, preventing apoptosis. BCL2 also binds to BECN1, preventing the assembly of the pre-autophagosomal structure, which results in the inhibition of autophagy. Furthermore, aberrant blockade of cell death by BCL2 overexpression has already been reported as a hallmark of high-risk MDSs [43]. In our case, this role of BCL2 in autophagy inhibition in MDSs could be indirectly confirmed through the low protein expression levels of the autophagic marker LC3B in both MDS risk groups.

Of particular interest was the recording of increased expression levels of the autophagic gene *TGM2* in MDS patients compared to healthy individuals, which was further confirmed at the protein level.

Recent studies showed that TGM2 can contribute to a variety of pathological functions through the deregulation of basic cellular processes, such as apoptosis, differentiation, and autophagy [44,45,46]. TGΜ2 may regulate the induction or inhibition of autophagy [47], either through cross-linking with members of the Beclin1 complex and/or by favoring the degradation of p53 in patients with pancreatic cancer [48]. In a recent study, it was demonstrated that protein kinase C-delta (PKCδ) constitutively protects cells from autophagy by upregulating TGM2 expression [49]. Another mechanism that has been proposed is the interaction of TGM2 with NF-κB, with the latter regulating autophagy induction in mantle cell lymphoma [50]. Furthermore, TGM2 can also serve as an anti-apoptotic or pro-apoptotic protein. Regarding the former, TGM2 catalyzes the polymerization of BAX, which results in the stabilization of a pore-forming and cytochrome c-releasing conformation [51]. Furthermore, silencing TGM2 could promote cell apoptosis through the upregulation of CASP3 and TIMP1 [52]. In contrast, TG2 may enhance EGF receptor expression in glioblastomas, inducing cell transformation [53]. Altogether, the elevated levels of *TGM2* observed in our study suggest that it could serve as a therapeutic target in MDSs.

Our study is limited by the comparison between BM samples from MDS patients and PB samples from healthy individuals, as well as the rather small size of our cohort. For the complete characterization of the role of autophagy and apoptosis in MDSs, further validation in larger series of samples is necessary, especially regarding experiments at the protein level. However, our study still has value, being the first to implement a systematic analysis of the expression patterns of a large series of genes implicated in autophagy and apoptosis in MDSs. We report evidence for the deregulation of these processes in MDS pathogenesis, being more pronounced in high-risk patients, as well as important information regarding the enhanced impact of autophagy on disease progression. Furthermore, we report here the first indications that TGM2 is overexpressed in both low- and high-risk groups of MDS patients, suggesting its potential role as a therapeutic target.

## 5. Conclusions

Our systematic analysis of the mechanisms of autophagy and apoptosis in MDSs revealed a clear downregulation. This downregulation pattern was observed in a large series of genes involved in both processes in MDSs compared to healthy donors, which was more evident in high-risk patients. These findings indicate the existence of defects in both mechanisms that become more pronounced along the natural history of MDSs. The decreased activation of autophagy and apoptosis probably enhances cell survival in the MDS cells. Furthermore, the higher level of downregulation observed in patients with high-risk MDSs could perhaps indicate the relevance of these two processes in disease progression. Finally, TGM2 was found to be overexpressed in both MDS groups. This may aid tumor growth by promoting the survival of the malignant cells through the inactivation of autophagy and apoptosis. Hence, inhibiting TGM2 represents a realistic approach to reversing these processes in cancer cells.

Overall, findings from the present study are expected to: (i) provide better insight into the complexity of MDSs, (ii) advance our understanding regarding the role of autophagy and apoptosis in disease pathogenesis, and (iii) assist in the identification of novel therapeutic targets. Pharmacological modulation of autophagy and apoptosis could represent new important clinical potential as a therapeutic strategy to eradicate cancer. The induction of autophagy and apoptosis may be useful for cancer chemoprevention or to activate an alternative cell death mechanism in malignant cells.

## Figures and Tables

**Table 1:** Basic clinical and biological characteristics of the MDS cohort in the present study according to the IPSS-R scoring system.

Characteristics	Patient Cohort
Median age at diagnosis, years (range)	75 (59–90)
Gender (male: female)	25:9 (2.77)
IPSS-R category, (*n*%)	
Very low	5.9
Low	32.4
Intermediate	0
High	44.1
Very high	17.6

**Table 2:** Expression analysis of 15 genes related to autophagy and apoptosis in patients with MDSs versus healthy controls with qRT-PCR.

	All MDS		Lower-Risk MDS		Higher-Risk MDS	
Gene Symbol	Fold Change	*p*-Value	Fold Change	*p*-Value	Fold Change	*p*-Value
*AMBRA1*	−3.01	0.791	−1.92	0.460	−4.26	0.168
*PI3KC3*	−4.97	0.001	−3.25	0.003	−6.91	0.001
*UVRAG*	−3.68	0.001	−3.11	0.012	−4.19	0.003
*ATG5*	−4.72	0.001	−4.46	0.002	−4.94	0.001
*ATG12*	−2.06	0.855	−1.41	0.508	−2.75	0.027
*ATG16L1*	−6.08	0.008	−4.90	0.005	−7.18	0.059
*DRAM1*	−5.64	0.001	−5.21	0.001	−6.00	0.016
*DRAM2*	−2.49	0.284	−2.12	0.845	−2.81	0.003
*MAP1LC3B*	−4.62	0.025	−3.99	0.0430	−5.16	0.054
*TGM2*	1.81	0.369	1.52	0.274	2.12	0.286251
*BCL2*	8.21	0.224	21.83	0.096	3.87	0.248
*CASP3*	−11.91	0.015	−6.92	0.053	−18.07	0.064
*CASP8*	−18.10	0.027	−3.77	0.166	−60.51	0.088
*CTSB*	−10.42	0.004	−4.91	0.068	−18.59	0.022
*CASP7*	−4.89	0.002	−1.98	0.090321	−9.78	0.007

## Data Availability

The data generated in the context of the study are openly available in GEO database at [https://www.ncbi.nlm.nih.gov/geo/query/acc.cgi?acc=GSE204797], reference number [GSE204797], accessed on 26 May 2022.

## Supplementary Materials

The following supporting information can be downloaded at: https://www.mdpi.com/article/10.3390/cimb45050263/s1, Table S1: Clinical and biological characteristics for each individual patient of the study cohort according to the IPSS-R scoring system, Table S2: Categorization of the 84 genes included in the Human Autophagy RT^2^ Profiler™ PCR Array according to their implication in the processes of autophagy, apoptosis and cell cycle regulation, Table S3: Primers sequences (5′–3′) used in the qPCR experiments, Table S4: Standard deviation values for samples from each analyzed group per individual gene, Table S5: Expression profiling of genes related directly or indirectly to autophagy in different groups of patients with MDS versus healthy controls by using a PCR array, Table S6: Standard Deviation of DCt (Ct gene of interest-Ct housekeeping gene) in PB samples versus paired BM samples from the same patients with MDS.
