# Supplementary material for: Deregulation of Autophagy and Apoptosis in Patients with Myelodysplastic Syndromes: Implications for Disease Development and Progression
Source: Curr Issues Mol Biol. 2023 May 8;45(5):4135–50. doi: 10.3390/cimb45050263 (PMC10217762; doi:10.3390/cimb45050263)
Supplement: Supplementary file 1 [file cimb-45-00263-s001.zip › cimb-2331264-supplementary.pdf]

Supplementary material

Materials and Methods

Real-time PCR-based array analysis

Regarding the processing of RNA samples, cDNA was mixed with the RT^2 SYBR Green Mastermix and RNase-free water according to the protocol. The qRT-PCR was performed on a BioRad CFX96 according to the RT^2 Profiler PCR Array instructions. The data were analyzed using the PCR Array Data Analysis QIAGEN web portal at GeneGlobe. Microarray expression data was obtained for each individual gene and was normalized against the housekeeping gene *HPRT1* by calculating the ΔCt. Fold changes in gene expression were generated using the $2^{-\Delta\Delta CT}$ method, with the cut-offs for significant upregulation and downregulation being set to >2 and <-2, respectively.

Supplementary Tables

Supplementary Table S1. Clinical and biological characteristics for each individual patient of the study cohort according to the IPSS-R scoring system.

IPSS-R category	Gender	Age at diagnosis
Very low	M	59
Very low	F	70
Low	M	72
Low	M	73
Low	M	76
Low	M	82

Low	M	83
Low	M	82
Low	M	80
Low	M	73
Low	F	65
Low	M	64
Low	F	78
High	M	88
High	M	83
High	F	89
High	F	70
High	M	90
High	M	86
High	M	66
High	M	64
High	F	70
High	M	60
High	M	80
High	M	76
High	M	82
High	F	78

High	F	79
Very high	M	67
Very high	M	62
Very high	M	62
Very high	M	66
Very high	M	85
Very high	F	90

Supplementary Table S2. Categorization of the 84 genes included in the Human Autophagy RT[2] Profiler™ PCR Array according to their implication in the processes of autophagy, apoptosis and cell cycle regulation.

Gene	Full gene name	Function
AMBRA1	Autophagy/beclin-1 regulator 1	autophagic vesicle formation
ATG12	ATG12 autophagy related 12 homolog (S. cerevisiae)	autophagic vesicle formation
ATG16L1	ATG16 autophagy related 16-like 1 (S. cerevisiae)	autophagic vesicle formation
ATG3	ATG3 autophagy related 3 homolog (S. cerevisiae)	autophagic vesicle formation
ATG4A	ATG4 autophagy related 4 homolog A (S. cerevisiae)	autophagic vesicle formation
ATG4B	ATG4 autophagy related 4 homolog B (S. cerevisiae)	autophagic vesicle formation
ATG4C	ATG4 autophagy related 4 homolog C (S. cerevisiae)	autophagic vesicle formation
ATG4D	ATG4 autophagy related 4 homolog D (S. cerevisiae)	autophagic vesicle formation
ATG5	ATG5 autophagy related 5 homolog (S. cerevisiae)	autophagic vesicle formation
ATG7	ATG7 autophagy related 7 homolog (S. cerevisiae)	autophagic vesicle formation
ATG9A	ATG9 autophagy related 9 homolog A (S. cerevisiae)	autophagic vesicle formation
ATG9B	ATG9 autophagy related 9 homolog B (S. cerevisiae)	autophagic vesicle formation
BECN1	Beclin 1, autophagy related	autophagic vesicle formation
GABARAP	GABA(A) receptor-associated protein	autophagic vesicle formation
GABARAPL1	GABA(A) receptor-associated protein like 1	autophagic vesicle formation
GABARAPL2	GABA(A) receptor-associated protein-like 2	autophagic vesicle formation
IRGM	Immunity-related GTPase family, M	autophagic vesicle formation
MAP1LC3A	Microtubule-associated protein 1 light chain 3 alpha	autophagic vesicle formation
MAP1LC3B	Microtubule-associated protein 1 light chain 3 beta	autophagic vesicle formation
RGS19	Regulator of G-protein signaling 19	autophagic vesicle formation
ULK1	Unc-51-like kinase 1 (C. elegans)	autophagic vesicle formation

CTSD	Cathepsin D	autophagosome-lysosome fusion
CTSS	Cathepsin S	autophagosome-lysosome fusion
DRAM1	DNA-damage regulated autophagy modulator 1	autophagosome-lysosome fusion
DRAM2	DNA-damage regulated autophagy modulator 2	autophagosome-lysosome fusion
LAMP1	Lysosomal-associated membrane protein 1	autophagosome-lysosome fusion
NPC1	Niemann-Pick disease, type C1	autophagosome-lysosome fusion
TMEM74	Transmembrane protein 74	autophagosome-lysosome fusion
HSP90AA1	Heat shock protein 90kDa alpha (cytosolic), class A member 1	Chaperone-mediated autophagy
HSPA8	Heat shock 70kDa protein 8	Chaperone-mediated autophagy
EIF4GI	Eukaryotic translation initiation factor 4 gamma, 1	intracellular signals of induced autophagy
ESR1	Estrogen receptor 1	intracellular signals of induced autophagy
GAA	Glucosidase, alpha; acid	intracellular signals of induced autophagy
HGS	Hepatocyte growth factor-regulated tyrosine kinase substrate	intracellular signals of induced autophagy
MAPK14	Mitogen-activated protein kinase 14	intracellular signals of induced autophagy
PI3KC3	Phosphoinositide-3-kinase, class 3	intracellular signals of induced autophagy
PIK3R4	Phosphoinositide-3-kinase, regulatory subunit 4	intracellular signals of induced autophagy
RPS6KB1	Ribosomal protein S6 kinase, 70kDa, polypeptide 1	intracellular signals of induced autophagy
ULK2	Unc-51-like kinase 2 (C. elegans)	intracellular signals of induced autophagy
UVRAG	UV radiation resistance associated gene	intracellular signals of induced autophagy
WIPI1	WD repeat domain, phosphoinositide interacting 1	protein targeting in autophagy
SQSTM1	Sequestosome 1	protein targeting in autophagy
ATG10	ATG10 autophagy related 10 homolog (S. cerevisiae)	protein transporters
ATG16L2	ATG16 autophagy related 16-like 2 (S. cerevisiae)	protein transporters
RAB24	RAB24, member RAS oncogene family	protein transporters
AKT1	V-akt murine thymoma viral oncogene homolog 1	apoptosis regulators
APP	Amyloid beta (A4) precursor protein	apoptosis regulators
BAD	BCL2-associated agonist of cell death	apoptosis regulators
BAK1	BCL2-antagonist/killer 1	apoptosis regulators

BAX	BCL2-associated X protein	apoptosis regulators
BCL2	B-cell CLL/lymphoma 2	apoptosis regulators
BCL2L1	BCL2-like 1	apoptosis regulators
BID	BH3 interacting domain death agonist	apoptosis regulators
BNIP3	BCL2/adenovirus E1B 19kDa interacting protein 3	apoptosis regulators
CASP3	Caspase 3, apoptosis-related cysteine peptidase	apoptosis regulators
CASP8	Caspase 8, apoptosis-related cysteine peptidase	apoptosis regulators
CLN3	Ceroid-lipofuscinosis, neuronal 3	apoptosis regulators
CTSB	Cathepsin B	apoptosis regulators
CXCR4	Chemokine (C-X-C motif) receptor 4	apoptosis regulators
DAPK1	Death-associated protein kinase 1	apoptosis regulators
EIF2AK3	Eukaryotic translation initiation factor 2-alpha kinase 3	apoptosis regulators
FADD	Fas (TNFRSF6)-associated via death domain	apoptosis regulators
FAS	Fas (TNF receptor superfamily, member 6)	apoptosis regulators
HDAC1	Histone deacetylase 1	apoptosis regulators
HDAC6	Histone deacetylase 6	apoptosis regulators
HTT	Huntingtin	apoptosis regulators
IGF1	Insulin-like growth factor 1 (somatomedin C)	apoptosis regulators
INS	Insulin	apoptosis regulators
MAPK8	Mitogen-activated protein kinase 8	apoptosis regulators
MTOR	Mechanistic target of rapamycin (serine/threonine kinase)	apoptosis regulators
NFKB1	Nuclear factor of kappa light polypeptide gene enhancer in B-cells 1	apoptosis regulators
PI3KCG	Phosphoinositide-3-kinase, catalytic, gamma polypeptide	apoptosis regulators
PRKAA1	Protein kinase, AMP-activated, alpha 1 catalytic subunit	apoptosis regulators
SNCA	Synuclein, alpha (non A4 component of amyloid precursor)	apoptosis regulators
TGM2	Transglutaminase 2 (C polypeptide, protein-glutamine-gamma-glutamyltransferase)	apoptosis regulators
TNF	Tumor necrosis factor	apoptosis regulators
TNFSF10	Tumor necrosis factor (ligand) superfamily, member 10	apoptosis regulators
TP53	Tumor protein p53	apoptosis regulators
CDKN1B	Cyclin-dependent kinase inhibitor 1B (p27, Kip1)	cell cycle regulators
CDKN2A	Cyclin-dependent kinase inhibitor 2A (melanoma, p16, inhibits CDK4)	cell cycle regulators
IFNG	Interferon, gamma	cell cycle regulators
PTEN	Phosphatase and tensin homolog	cell cycle regulators
RB1	Retinoblastoma 1	cell cycle regulators
TGFB1	Transforming growth factor, beta 1	cell cycle regulators

Supplementary Table S3. Primers sequences (5′ – 3′) used in the qPCR experiments.

Gene	Direction	Sequence
ATG5	Forward	CCCATCAATCGGAAACTCATG
	Reverse	CCCCATCTTCAGGATCAATAGC
ATG12	Forward	TTTGCTAAAGGCTGTGGG
	Reverse	AAGGAGCAAAGGACTGAT
ATG16	Forward	TGCAGTTCAGTCCAGGTTC
	Reverse	GCTAAGAGGTAAGATCCAGCAC
DRAM1	Forward	TGGGATGTTTCGGAATGGG
	Reverse	AAGAAACGGCAGAGATGACC
DRAM2	Forward	ATGTAAGTGGAGCTGTGCTTACCTTTGGT
	Reverse	ACTGTGCAAAACTGATGAGCAAGTCAG
AMBRA1	Forward	AACCCTCCACTGCGAGTTGA
	Reverse	TCTACCTGTTCCGTGGTTCTCC
UVRAG	Forward	AAACTGACGGAAAAGGAGAG
	Reverse	CCATGTTGATATCTTAGCTGTGC
PI3K	Forward	GGGATTAGTGCTGAGGTCATG
	Reverse	AGTCTATGTGGAAGAGTTTGCC
TGM2	Forward	CCAACTACAACTCGGCCCAT
	Reverse	CTGGTCATCCACGACTCCAC
LC3B	Forward	GAGAAGCAGCTTCCTGTTCTGG
	Reverse	GTGTCCGTTCACCAACAGGAAG
BCL2	Forward	CGGAGGCTGGGATGCCTTTG
	Reverse	TTTGGGGCAGGCATGTTGAC
CASP3	Forward	TGGTTTGAGCCTGAGCAGAGA
	Reverse	TCATCCACACATACCAGTGCG
CASP7	Forward	TCTTCGGCCTATTCCACGGTTC
	Reverse	TTCCAGGTCTTTTCCGTGCTC
CASP8	Forward	AAGGAGCTGCTCTTCCGAATT
	Reverse	AATTTGAGCCCTGCCTGGT
CTSB	Forward	TGTAATGGTGGCTATCCTGCT
	Reverse	AGGCTCACAGATCTTGCTACA
HPRT1	Forward	TGACACTGGCAAAACAATGCA
	Reverse	GGTCCTTTTCACCAGCAAGCT

Supplementary Table S4. Standard deviation values for samples from each analyzed group per individual gene.

Standard Deviation of average ΔCt			
	Controls	Lower-risk MDS	Higher-risk MDS
ATG12	0.12	0.06	0.23
DRAM1	0.18	0.12	0.06
DRAM2	0.21	0.08	0.23
ATG16	0.09	0.23	0.18
PI3KC3	0.06	0.21	0.08
ATG5	0.04	0.15	0.23
AMBRA1	0.23	0.12	0.21
UVRAG	0.15	0.04	0.18
TGM2	0.17	0.23	0.23
LC3B	0.11	0.24	0.09
CASP7	0.12	0.13	0.25
CASP8	0.19	0.09	0.12
CASP3	0.13	0.06	0.14
CTSB	0.12	0.12	0.21
BCL2	0.08	0.02	0.22

Supplementary Table S5. Expression profiling of genes related directly or indirectly to autophagy in different groups of patients with MDS versus healthy controls by using a PCR array.

	**Fold change**		
**Gene**	**All MDS**	**Lower-risk MDS**	**Higher-risk MDS**
AMBRA1		-2.8	
AKT1	-6.73		-20.66
APP			-13.83
ATG10		-3.17	
ATG12	-4.95		-10.97
ATG16L1	-4.56	-3.52	
ATG16L2	-10.74		-27.42
ATG3	-5.81		-18.58
ATG4C		-2.65	
ATG4D	-3.83	-2.66	
ATG5	-3.5		-6.53
ATG7			-7.76
ATG9A		-3.38	
ATG9B		-5.71	
BAD	-3.57	-2.58	
BAX	-6.49	-2.51	-16.76

BCL2		-4.29	
BCL2L1		-3.14	
BECN1	-7.96	-4.1	-15.46
BID	-10.87	-2.95	-40
BNIP3	-5.88	-3.15	-10.97
CASP3	-6.07	-2.33	-15.85
CASP8	-16.09	-6.07	-42.68
CDKN1B	-9.17	-3.65	-23.02
CDKN2A		-3.26	
CLN3		-2.98	
CTSB	-6.63	-2.92	-15.05
CTSD			-32.37
CTSS	-36.3	-5.92	-222.55
CXCR4			-61.59
DAPK1		-6.36	
DRAM2	-5.27	-2.5	-11.12
EIF2AK3		-2.48	
EIF4G1			-13.92
ESR1		-4.64	

FADD	-5.55	-3.53	-8.74
FAS	-10.99	-3.79	-31.84
GAA	-8.34	-5.08	-13.69
GABARAP	-14.22	-3.2	-63.24
GABARAPL1	-11.4	-5.02	-25.91
GABARAPL2	-14.21	-3.68	-54.85
HDAC1	-7.14	-2.5	-20.38
HGS		-6.29	
HSPA8	-11.48	-3.2	-41.24
IGF1		-2.86	
INS		-4.73	
IRGM		-6.34	
LAMP1	-6.66	-3.97	
MAP1LC3A	-5.59	-4.43	-7.05
MAP1LC3B			-10.78
MAPK8	-3.53	-2.51	
MAPK14			-22.42
NFKB1	-4.82	-2.8	-8.32
NPC1	-4.93	-3.4	-7.13

PIK3C3	-4.18		-6.71
PIK3CG			-10.35
PRKAA1			-5.82
PTEN	-15.86	-4.13	-60.88
RB1	-4.45		-10.76
RGS19	-12.19	-4.87	-30.51
SQSTM1	-12.59	-3.47	-45.68
TGFB1	-19.55	-5.11	
TMEM74		-6.34	
TNF		-4.78	
TNFSF10	-17.97	-5.36	-60.19
TP53	-5.34		-15.07
ULK1		-4.63	
UVRAG		-2.22	

The table presents all genes with ≥2 (mean value) fold change (up- or downregulated) and statistical significance of $p<0.05$ as identified by the PCR array data analysis in different (sub)groups of MDS samples compared to controls. The *HPRT1* gene was used as internal standard. Genes are sorted in alphabetical order.

Supplementary Table S6. Standard Deviation of DCt (Ct gene of interest- Ct housekeeping gene) in PB samples versus paired BM samples from the same patients with MDS.

	ATG12	DRAM1	DRAM2	ATG16	PI3K	ATG5	AMBRA	UVRAG	TGM2	BCL2	CASP7	LC3B
**Patient 1**	0.58	0.61	0.16	0.62	0.5	0.32	0.62	0.18	0.10	0.72	1.11	0.30
**Patient 2**	0.35	0.12	0.45	0.52	0.15	0.23	0.16	0.69	0.13	0.13	0.13	0.13
**Patient 3**	0.45	0.12	0.27	0.63	0.14	0.42	0.21	0.34	0.69	0.47	0.23	0.67
